# Investigation of *Arabidopsis* root skototropism with different distance settings

**DOI:** 10.1080/15592324.2024.2348917

**Published:** 2024-05-05

**Authors:** Xingyu Yan, Yongshun Liang, Felipe Yamashita, František Baluška

**Affiliations:** Institute of Cellular and Molecular Botany, University of Bonn, Bonn, Germany

**Keywords:** Abiotic stress, glutamate-like receptor, PIN proteins, root tropism, light conditions

## Abstract

Plants can activate protective and defense mechanisms under biotic and abiotic stresses. Their roots naturally grow in the soil, but when they encounter sunlight in the top-soil layers, they may move away from the light source to seek darkness. Here we investigate the skototropic behavior of roots, which promotes their fitness and survival. Glutamate-like receptors (GLRs) of plants play roles in sensing and responding to signals, but their role in root skototropism is not yet understood. Light-induced tropisms are known to be affected by auxin distribution, mainly determined by auxin efflux proteins (PIN proteins) at the root tip. However, the role of PIN proteins in root skototropism has not been investigated yet. To better understand root skototropism and its connection to the distance between roots and light, we established five distance settings between seedlings and darkness to investigate the variations in root bending tendencies. We compared differences in root skototropic behavior across different expression lines of *Arabidopsis thaliana* seedlings (*atglr3.7 ko, AtGLR3.7 OE*, and *pin2 knockout*) to comprehend their functions. Our research shows that as the distance between roots and darkness increases, the root’s positive skototropism noticeably weakens. Our findings highlight the involvement of GLR3.7 and PIN2 in root skototropism.

## 1. Introduction

To adapt to various environments (e.g., freezing conditions, dry conditions, light environments, etc.), each living organism has to respond properly to its surroundings. Plants cannot move away from extremes in their environment, but they do have their own adaptive adjustment mechanisms, modifying their developmental architecture or behavioral characteristics to cope with environmental stresses. In a general plant life cycle, there are six typical stages involved, including seed germination, vegetative development, inflorescence development, inflorescence, fertilization, and ripening.^1^ After seed germination, one of the first organs that start to develop is the roots.

Roots are the underground part of the plant body and are required for anchorage in the substrate, water and ions uptake, phytohormones synthesis, nutrient storage, vegetative growth, etc. The root apex is subdivided into four zones: meristematic, transition, elongation, and differentiation zones^2,3^). Also at the root apex, can be found the root cap, responsible for sensing the gravity pull, protects the root apical meristem (RAM) from physical damage (such as stones), and controls the root’s downward growth.^4^ This downward root growth is carried out by specialized cells of the root cap, called statocytes. Statocytes are cells that contain amyloplasts, plastids filled with starches, which sediment in the lower part of the cells, hence allowing the root to be reoriented. The cells in the elongation zone elongate, allowing root growth.^5^ One of the main growth-related phytohormones is auxin, which is crucial for cell elongation and lateral root growth^4^ and light plays a key role in auxin production and transport.^6,7^

One of the most important environmental factors for plant growth and development throughout their life cycle is light. For example, light controls seed germination, plant development, flowering, and metabolism.^8^ Light also provides energy for both photosynthesis and photomorphogenesis, two mechanisms that determine plant growth. Light sensing is an essential factor for plants and changes in intensity and quality cause plants to change their morphological traits.^9^ Besides that, light also directs the movement of plant organs in a specific direction, which is defined as phototropism, as early described by Charles Darwin.^10^ This dynamic plant growth and morphogenesis is mainly under control of the plant hormone auxin.^11^

The asymmetric distribution of auxin causes the cells on the plant’s darker side to elongate, leading the plant to bend toward the light source. Under normal physiological conditions, a significant proportion of apoplastic auxin exists in its protonated form, indole-3-acetic acid (IAAH), which can freely permeate cell membranes. This process is facilitated by members of the AUXIN/LIKE AUX1 (AUX/LAX) family of auxin importers. Upon entering the cell, where the intracellular pH is neutral, the weak acid form of auxin, indole-3-acetate (IAA^−^), becomes trapped and necessitates the activity of efflux carriers for extrusion, allowing for intercellular transport.^12^ Two families of transporters are involved in this process. The long-PINs, including PIN1–4 and PIN7 in *Arabidopsis thaliana*, serve as efflux carriers responsible for the directional movement of auxin between cells. Additionally, the ATP-binding cassette B (ABCB) class, comprising several multi-drug resistance transporters, also participates in auxin efflux and facilitates intercellular transport of auxin.^13^ Moreover, the phototropin (PHOT) blue light receptors are important,^14^ interacting with the PIN2 in light-induced root responses.^15^

In *Arabidopsis* and other flowering plants, there are two PHOTs present, namely phot1 and phot2. Phot1 primarily acts as the photoreceptor for root phototropism and hypocotyl phototropism across a wide range of blue light intensities. In contrast, the involvement of phot2 in hypocotyl phototropism is limited to high light intensities. This restriction is mainly attributed to the increase in protein abundance of phot2 mediated by light exposure.^16^ Interestingly, this phototropic response is observed across a wide range of light intensities, spanning from very low levels of light to the intensity of blue light experienced on a sunny day.^17^

In contrast to phototropism, skototropism is the term given to growth or movement of plant organs toward the darkness,^18^ emphasizing the movement of roots seeking darkness. Roots normally grow downwards, following the gravity vector.^19^ However, under natural conditions, roots can encounter sunlight in the soil’s upper layers. Once this happens, they bend or stretch away from the light source to search for darkness, which allows them to avoid exposure to potentially unfavorable light conditions. Unfortunately, roots of seedlings grown in the transparent Petri dishes are exposed to strong light causing seedling stress and altered seedling morphogenesis and root-shoot ratio.^20,21^

According to Gottlieb Haberlandt’s^22^ hypothesis of plant ocelli, the upper epidermal cells of leaves are shaped like convex or Plano convex lenses.^23^ By gathering light rays together, these “lenses” allow light-sensitive epidermal cells to recognize the size and shape of other plants in their surroundings. In addition to the leaves, the root apex may also have ocelli, since the roots can adapt to lower levels of light in the soil. The major factors that influence the negative phototropic response in plant roots are primarily the blue light signal and the activity of the PHOT blue light receptors.^24,25^ In roots, the presence of blue light triggers a signaling cascade inducing root growth away from the light source (negative phototropism), a response mediated by the PHOT1 receptor.^15,26^

Calcium (Ca^2+^), a key second messenger in plant cells, plays an important role in signaling responses to environmental changes. To produce free cytosolic Ca^2+^ transients, Ca^2+^ permeable channels, such as GLRs,^27^ must be opened to control the influx of cytosolic Ca^2+^.^28^ In animals, one important channel responsible for cytosolic Ca^2+^ is the ionotropic glutamate receptor channels (iGluRs). The opening of iGluR allows glutamate entrance into the postsynaptic neuron and allows calcium (Ca^2+^) transport.^29^ Glutamate is involved in signal transmission between neurons, particularly at synapses. It has been extensively studied and recognized as a fundamental signaling molecule in animals for more than five decades.^30^ It is essential for cognitive functions, learning, memory, and various other important biological processes. Moreover, glutamate, which is synthesized by the enzyme glutamate synthase using the substrates glutamine and 2-oxoglutarate, plays a crucial role in the metabolism of amino acids in plants.^31^

Since the discovery of the 20 genes in *Arabidopsis* as homologs of iGluRs, it has led to extensive research on these genes in plants.^32^ Plant glutamate-receptor-like receptors (GLRs) exhibit significant similarity to their animal counterparts in terms of their nucleotide and amino acid sequences.^33^ While iGluRs mediate neurotransmission in mammals, GLRs in plants serve crucial roles in various plant-specific physiological processes such as stress response and adaptation, sexual reproduction, pollen tube growth, stomata aperture regulation, innate immune and wound responses.^27–36^ One of their key functions is the regulation of Ca^2+^ signaling. In the presence of specific amino acids, GLRs can facilitate the movement of various cations, including Ca^2+^, across the cell membrane and into the cytoplasm.^37,38^ This influx of Ca^2+^ acts as a major signaling player within the cell, having a vital role in intracellular signaling pathways in plants. GLRs in *Arabidopsis*, known as AtGLRs, serve as both sensors and mediators for a wide range of external and internal signals in plants.

Despite enormous advancements in the comprehension of the function of GLR in plants, the understanding of the biological function of these receptors is still in a stage of development. Therefore, the aim of this study was to demonstrate the skototropic root behavior of *Arabidopsis* seedlings positioned at different distances from darkness (0, 10, 20, 30, and 40 mm), including wild-type (Col-0), AtGLR3.7 knockout line *(atglr3.7 ko)*, AtGLR3.7 over-expression line *(AtGLR3.7 OE)*, and AtPIN2 deletion mutants *(pin2 knockout)*.

## 2. Material and methods

### 2.1. Growth media preparation

The growth medium was prepared by mixing the Murashige and Skoog (MS) media salt (with vitamins), saccharose, and dH_2_O. After adding each to a 1 L container, the pH was adjusted to 5.8 using KOH or HCl. After that, 4 g of phytagel was added to the prepared mixed solution of 1 L. The medium was mixed and autoclaved at 120°C. The medium was placed in Petri dishes of different sizes and prepared under a sterile bench for further usage.

### 2.2. Seeds preparation

All plant genotypes used in this study had the background of *Arabidopsis thaliana* Col-0. The AtGLR3.7 knockout line (*atglr3.7 ko*) was kindly provided by Prof. Lai-Hua Liu (China Agricultural University, Beijing, China). The AtGLR3.7 over-expression line (*AtGLR3.7 OE*) was provided by Dr. Matthias Weiland, a former student at our laboratory (Institute of Cellular & Molecular Botany, University of Bonn, Bonn, Germany). The AtPIN2 deletion line (*pin2 knockout*) and *Arabidopsis* wild-type (Col-0) seeds were ordered from the European Arabidopsis Stock Centre (Nottingham, United Kingdom). Sterile growth conditions were maintained by surface sterilization of *Arabidopsis* seeds. Rough sterilization was done in 70% ethanol for 3 min, followed by sodium hypochlorite solution for 5 min. Seeds were washed five times in distilled water. Sterilized seeds were sown on square Petri dishes with ½ MS medium under the sterile bench. Petri dishes with sterilized seeds were stored in the fridge for stratification for 48 h at 4°C and transferred to the growth chamber for 36 h for seed germination. The conditions of the growth chamber were as follows: the temperature was 17–24°C, and the light intensity was 121.43 μmol s^−1^ m^−2^.

### 2.3. Skototropism experimental preparation

**Figure 1.**
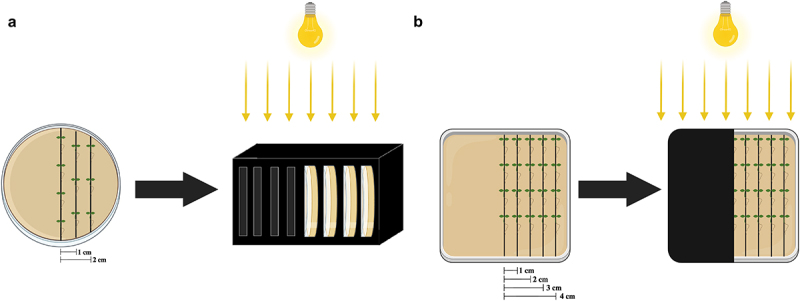
Experimental setup in this study. Examples of different shade approaches we adopted: (a) treatment of small round Petri dishes (92 × 16 mm) within black boxes with a light source at the growth chamber ceiling. (b) Treatment of large square Petri dishes darkened with black covers with a light source at the growth chamber ceiling. Based on the sizes of the petri dishes, three rows of *Arabidopsis* seedlings were positioned in a and five rows in B, respectively. Each column was spaced 1 cm (10 mm) apart from each other as the label. To ensure consistent positioning, the inner row of seedlings was aligned with the border of the covers.

To investigate the influence of distance to darkness on *A. Thaliana* roots for the skototropism experiment, seedlings were transferred to various-sized Petri dishes. After 36 h for seed germination, seedlings were transferred to new Petri dishes, according to the treatment, and placed in a vertical position, one below the other with straightened roots. Depending on the different sizes of the Petri dishes, the *Arabidopsis* seedlings were put in three or five columns, resulting in settings with various distance patterns (0, 10, 20, 30, and 40 mm) from the seedlings to the darkness (Figure 1). After placing the seedlings, all dishes were sealed with parafilm and then placed in construction that held one-half of the Petri dish in darkness or shaded with a black cover (Figure 1). Four groups of Petri dishes with shades were arranged as below: (A) Small round dishes (92 × 16 mm) with three columns of seedlings were inserted into black boxes, resulting in light intensity on the darkness side of 39.74 μmol s^−1^ m^−2^; (B) Small round dishes with three columns of seedlings were placed into black covers, resulting in light intensity on the darkness side of 15.34 μmol s^−1^ m^−2^; (C) Large round dishes (150 × 20 mm) with five columns of seedlings were placed into black covers, resulting in light intensity on the darkness side of 19.10 μmol s^−1^ m^−2^; (D) Square dishes (120 × 120 × 17 mm) with five columns of seedlings were placed into black covers, resulting in light intensity on the darkness side of 20.17 μmol s^−1^ m^−2^. Light source was at the growth chamber ceiling (Figure 1).

### 2.4. Measurements and evaluation

After 96 hours, the Petri dishes were scanned. The root bending angle was measured via Fiji ImageJ software based on digital images. The values for root bending were sorted into 3 groups: (1) Positive values, showing a bending toward darkness; (2) Negative values, indicating a bending away from darkness; (3) A group zero (180° ± 1°), exhibiting no visible behavior toward or away from light darkness. Statistical analysis, graphing, and data visualization were performed using GraphPad Prism (version 9.5.1) software.

## 3. Results

### 3.1. Treatment of small round Petri dishes (92 × 16 mm) within black boxes

**Figure 2.**
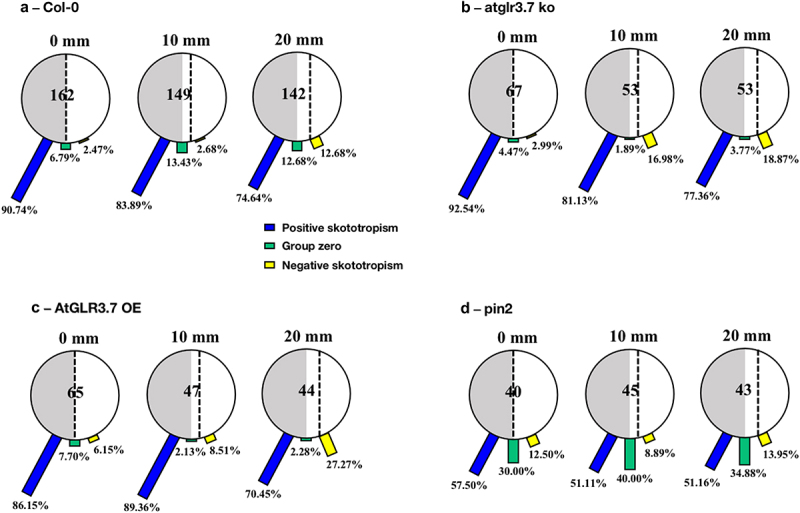
Skototropic response of *Arabidopsis* roots after 96 h growth within small round dishes inserted into the black box. Four lines of *Arabidopsis* seedlings were adopted: (a) *Arabidopsis thaliana* (col-0), (b) AtGLR3.7 knockout line (*atglr3.7 ko*), (c) AtGLR3.7 over-expression line (*AtGLR3.7 OE*), and (d) AtPIN2 deletion line (*pin2 knockout*). The circle contains the total number of *Arabidopsis* seedlings used in the experiment at the following distance settings: 0, 10, and 20 mm. The blue bars, green bars, and yellow bars, respectively, show the percentages of seedlings positively bending toward darkness, seedlings with no discernible bending trend, and seedlings bending away from darkness.

We found that for the Col-0 seedlings positioned on the borderline between light and darkness, 90.74% of them were bent to the darkness, implying a positive bending angle, after a 96-hour growing period. When the seedlings were placed on the light-biased side, 10 mm from the dividing line, 83.89% of them bent toward the darkness, and only 2.68% were grown toward the light. Further expanding the distance between Col-0 seedlings and the light-dark borderline to 20 mm, 74.64% of them were positively bent, 12.68% of them were bent to light, and 12.68% of them were grown without any preferred direction, growing downwards. Figure 2a shows a clear difference in root bending directions as the distance between the Col-0 seedlings and the light-dark borderline increases after 96 hours of growth.

For the seedlings of the *atglr3.7 ko*, there were 92.54% of them placed on the borderline between light and darkness (0 mm) bending to darkness. When the distance toward darkness increased to 10 mm, 81.13% of them bent toward darkness, and the other 16.98% bent away from the light. When it came to the distance of 20 mm to the darkness in the *atglr3.7 ko* line, there was a more noticeable increase in positive skototropism compared to the Col-0 line, reaching 77.36% (Figure 2b).

For the seedlings of the *AtGLR3.7 OE*, out of the 65 grown in the black box treatment for 96 hours at the border of darkness (0 mm), 86.15% bent toward the darkness, while 6.15% bent away from the darkness. The percentages of mutants bending toward darkness changed to 89.36% and 70.45% (Figure 2c) as the distances between the mutants and the darkness increased to 10 mm and 20 mm, whereas the proportions of those bending toward light rose to 8.51% and 27.27%, respectively.

Moreover, seedlings of AtPIN2 deletion lines (*pin2 knockout*) with a distance of 0 mm, 10 mm, and 20 mm from the light-dark borderline had almost the same proportion of bending to darkness, with bending to darkness proportions ranging from 50% to 60%. *Pin2* mutants that were 0 mm away from the borderline showed a proportion of bending to the darkness of 57.50%, which was slightly higher than seedlings in the other two circumstances (10 mm and 20 mm) (Figure 2d).

### 3.2. Treatment of small round Petri dishes (92 × 16 mm) darkened with black covers

**Figure 3.**
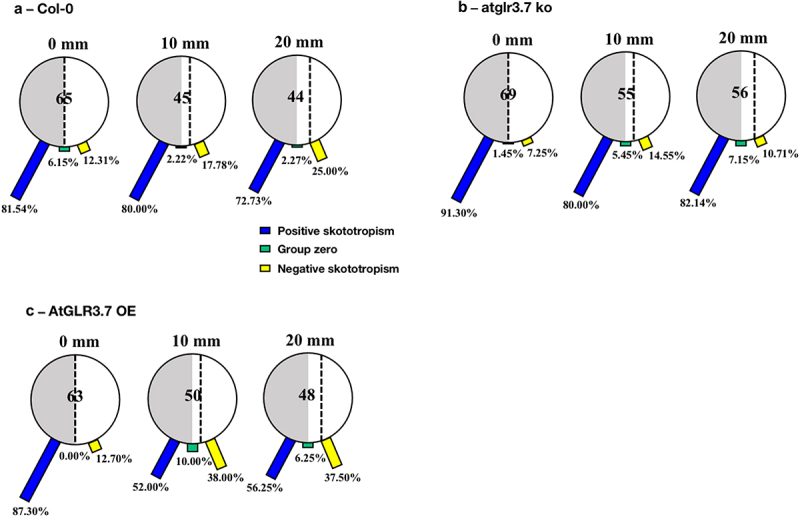
Skototropic response of *Arabidopsis* roots after 96 h growth with small round dishes darkened with black covers. Three lines of *Arabidopsis* seedlings were adopted: (a) *Arabidopsis thaliana* (col-0), (b) AtGLR3.7 knockout line (*atglr3.7 ko*), and (c) AtGLR3.7 over-expression line (*AtGLR3.7 OE*). The circle contains the total number of *Arabidopsis* seedlings used in the experiment at the following distance settings: 0, 10, and 20 mm. The blue bars, green bars, and yellow bars, respectively, show the percentages of seedlings positively bending toward darkness, seedlings with no discernible bending trend, and seedlings bending away from darkness.

The experimental results of Col-0 seedlings grown in the small round Petri dishes with black covers are shown in Figure 3a. Most of them (81.54%) were positioned at the border of darkness (0 mm), showing positive root skototropism with bending angles to darkness, while 12.31% of them bent away from darkness. Furthermore, Col-0 seedlings that were 10 mm and 20 mm away from the darkness-light borderline showed the proportions of bending to the darkness of 80.00% and 72.73%.

The *atglr3.7 ko* had larger percentages of root-positive skototropism than the Col-0 line at distances of 0, 10, and 20 mm from the light-dark borderline, exhibiting correspondingly 91.30%, 80.00%, and 82.14% (Figure 3b).

When the distances between the darkness-light borderline and seedlings were increased to 10 mm and 20 mm for the *AtGLR3.7 OE*, there was a significant decrease in the proportion of positive root skototropism compared to the other two lines, and the proportion of bending away from darkness increased sharply, reaching about 38% (Figure 3c).

### 3.3. Treatment of large round Petri dishes (150 × 20 mm) darkened with black covers

**Figure 4.**
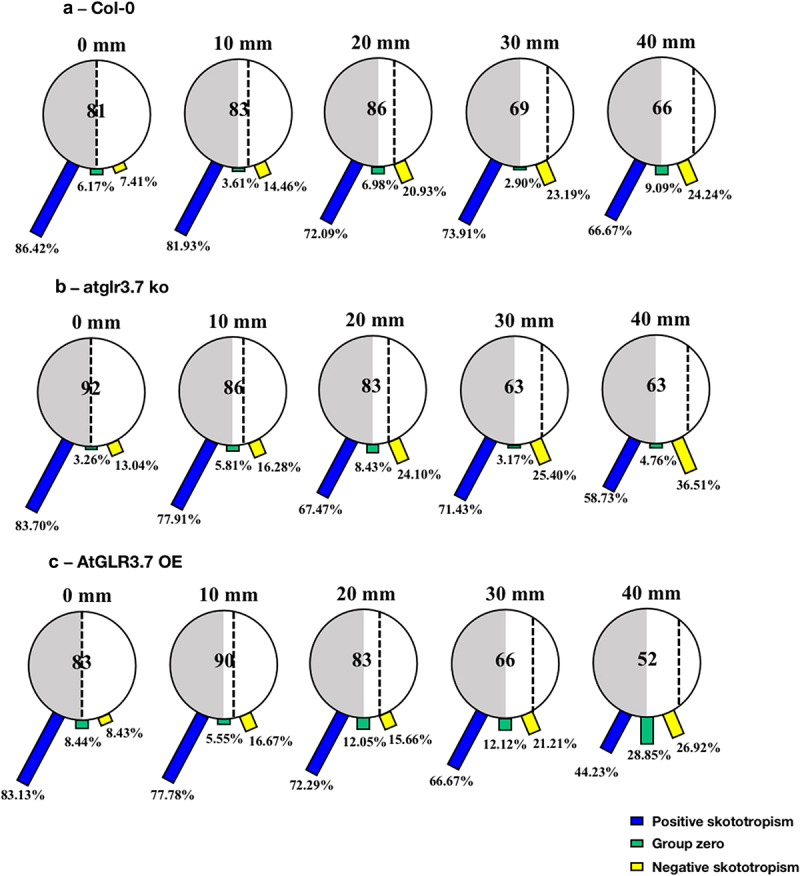
Skototropic response of *Arabidopsis* roots after 96 h growth with large round dishes partially covered with black covers. Three lines of *Arabidopsis* seedlings were adopted: (a) *Arabidopsis thaliana* (col-0), (b) AtGLR3.7 knockout line (*atglr3.7 ko*), and (c) AtGLR3.7 over-expression line (*AtGLR3.7 OE*). The circle contains the total number of *Arabidopsis* seedlings used in the experiment at the following distance settings: 0, 10, 20, 30, and 40 mm. The blue bars, green bars, and yellow bars, respectively, show the percentages of seedlings positively bending toward darkness, seedlings with no discernible bending trend, and seedlings bending away from darkness.

After the growth period of 96 h, with the increase of distances between Col-0 seedlings and the light-dark borderline from 0, 10, 20, 30, and 40 mm, the proportion of positive skototropism of Col-0 seedlings grown on black-covered large round Petri dishes showed a significant downward trend, with specific values of 86.42%, 81.93%, 72.09%, 73.91%, and 66.67%, and the portions of seedlings bending away from the light also increased in accordance (Figure 4a). The seedlings of the *atglr3.7 ko* and the *AtGLR3.7 OE* also showed root skototropic behavior consistent with the Col-0 line (Figures 4b,c).

### 3.4. Treatment of large square Petri dishes (120 × 120 × 17 mm) darkened with black covers

**Figure 5.**
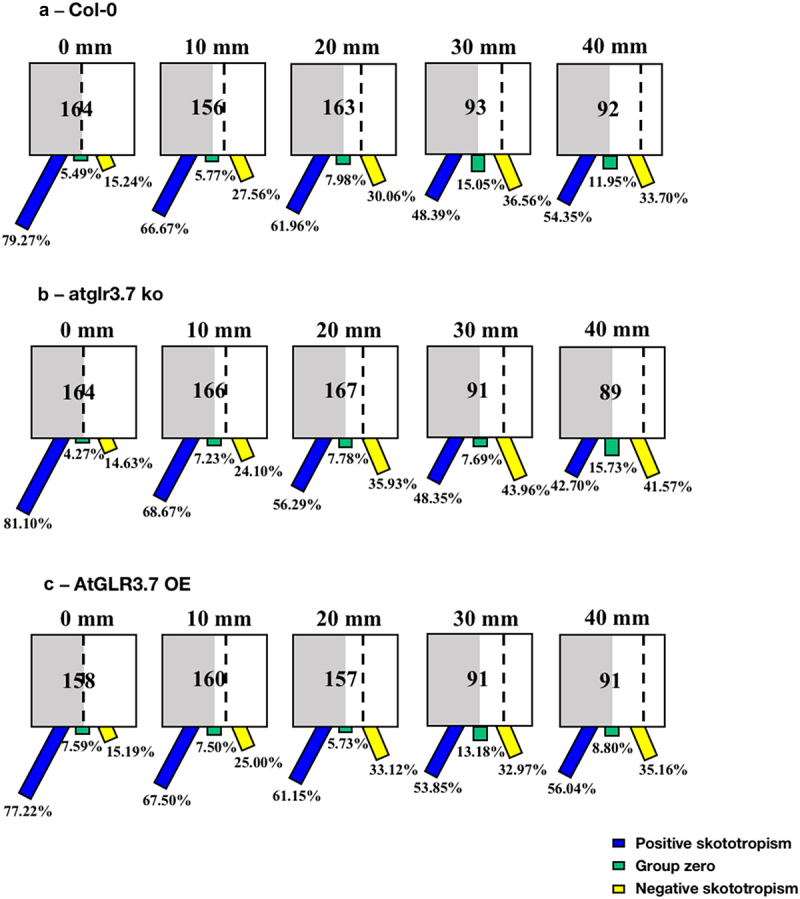
Skototropic response of *Arabidopsis* roots after 96 h growth with square dishes partially covered with black covers. Three lines of *Arabidopsis* seedlings were adopted: (a) *Arabidopsis thaliana* (col-0), (b) AtGLR3.7 knockout line (*atglr3.7 ko*), and (c) AtGLR3.7 over-expression line (*AtGLR3.7 OE*). The circle contains the total number of *Arabidopsis* seedlings used in the experiment at the following distance settings: 0, 10, 20, 30, and 40 mm. The blue bars, green bars, and yellow bars, respectively, show the percentages of seedlings positively bending toward darkness, seedlings with no discernible bending trend, and seedlings bending away from darkness.

When *Arabidopsis* seedlings were grown in square Petri dishes with black covers, the tendency of wild-type (Col-0), *atglr3.7 ko*, and *AtGLR3.7 OE* seedlings to bend to darkness reduced with the increase in distance between seedlings and darkness (Figure 5a–c). Importantly, for seedlings of the *atglr3.7 ko*, when the distance from the darkness reached 30 mm and 40 mm, the proportion of seedlings bent toward darkness (positive skototropism), and the proportion of seedlings bent away from darkness (negative skototropism) were nearly identical, approximately 40% (Figure 5b).

## 4. Discussion

**Figure 6.**
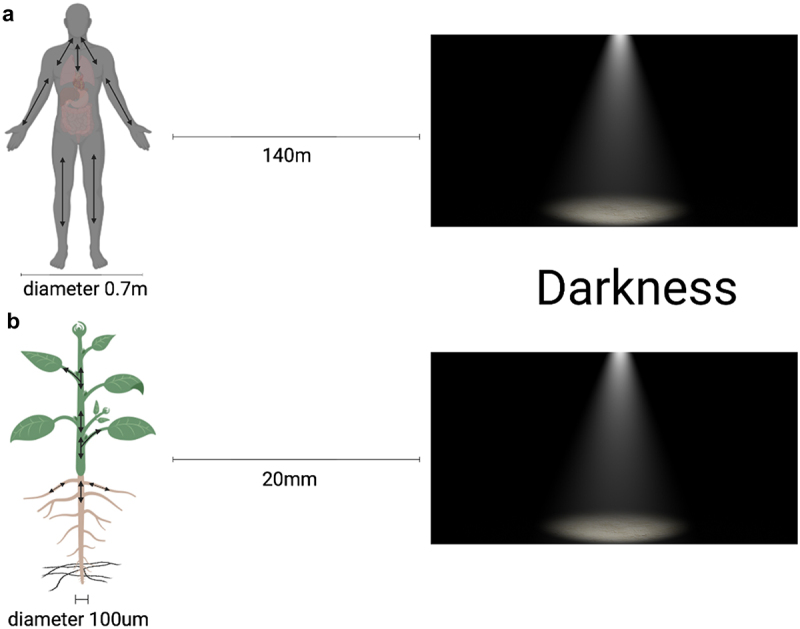
Comparative perception of darkness in *Arabidopsis* and human being. The *Arabidopsis thaliana* seedling (about 100 μm in diameter) positioned at 20 mm from darkness is equivalent to a person with a diameter of 0.7 m being situated 140 m away from darkness.

Even though plant roots develop in soil that is almost completely dark in nature, they are highly sensitive to light. Light stress conditions stimulate root growth as the roots try to escape light by increasing their growth rate, a strategy known as “root escape tropism”.^21^ The combination of light-induced root development and negative phototropism can be regarded as a physiologically relevant reaction since it induces light-exposed roots to return to the dark soil in nature.^21^ The analysis of our experimental data supports Yokawa’s study in that the general trend of decreasing skototropism with increasing distance to darkness remained consistent across the different cover treatments. This skototropic behavior is believed to be an adaptive mechanism that allows roots to avoid potentially unfavorable light conditions like in the upper layers of the soil. Furthermore, as shown in Figure 6, when the seedling with a diameter of 100 μm is 20 mm (2 cm) away from darkness, this corresponds to a person with a diameter of 0.7 m (70 cm) being 140 m away from the darkness (Figure 6). When the seedling is 10 mm (1 cm) away from the darkness, it is equivalent to 70 m away from the darkness. Plants are unable to sense dark surroundings as distances expand significantly, resulting in no escape tropism or behavior. Our experimental results also revealed that when the distance increased to 40 mm, the proportionate gap between positive and negative root skototropism decreased.

Despite the fact that almost all plant roots growing in nature are underground, in darkness, all photoreceptors are expressed at the root apices.^39^ Although a weak light is not stressful for the roots, they try to avoid strong lights. Recent studies have shown that *Arabidopsis* roots grew faster when grown in a light gradient environment, growing toward darkness. Based on this growth, one can imply some kind of vision through the root apex.^39–41^

The hypothesis that plants can have some sort of vision was first proposed by Gottlieb Haberlandt, in 1905 and called “Plant Ocelli”. He argued that the leaf epidermis can resemble a convex or Plano convex lens.^22^ Haberlandt’s theory was tested experimentally^42^ as well as supported by studies of a mimicking plant *Boquila trifoliolata*.^23,43,44^ This plant has the intriguing ability to change the shape of its leaves according to the host plant. When plastic leaves were presented to *Boquila trifoliolata*, it changed the shapes of leaves from three-lobed leaves to longitudinal leaves, mimicking the plastic leaves too.^45^

Parallel to the hypothesis of plant ocelli, the distribution pattern of phot1 in the transition zone of the root apex suggests a role for this region in blue light sensing, while the root cap is specialized for red light sensing.^39^ Recent research findings have demonstrated that red light and blue light can upregulate the transcription levels of several genes encoding GLR proteins. The transcriptional upregulation of AtGLRs under red light conditions is primarily regulated through pigment-mediated processes. The involvement of cryptochromes in this process is less evident, as some mutants show a significant reduction in red light induced AtGLRs transcriptional upregulation, while the high-level blue light upregulation by cryptochromes remains unaffected. These findings not only highlight the complex regulation of AtGLR upregulation but also suggest the possibility of AtGLR playing an important role in skototropism.^46^ According to our results, the *atglr3.7 ko* showed a higher proportion of positive skototropism compared to the wild-type (Col-0) line, which suggests that AtGLR3.7 may play a role in modulating the skototropic response in *Arabidopsis* roots. On the other hand, the *AtGLR3.7 OE* showed a decrease in positive skototropism and an increase in negative skototropism, indicating that overexpression of AtGLR3.7 may disrupt the normal skototropic response.

Moreover, the AtPIN2 deletion mutants (*pin2 knockout*) showed different skototropic behavior to the wild-type line (Col-0), the curvature of the root hardly changes according to the distance from darkness, implying that the PIN2 protein, which is involved in auxin transport, may play a major role in mediating the skototropic response in *Arabidopsis* roots. As mentioned, the localization of PIN proteins, responsible for polar auxin transport in the root apex, undergoes constant recycling between the plasma membrane and endosomal compartments.^47^ PIN2 protein has been identified to be involved in root negative phototropism.^48^ In dark-grown roots, PIN2 is not polarly localized at the plasma membrane but accumulates within endosomes/vacuoles.^48,49^ However, despite the absence of functional PIN2 protein, approximately 50% of the *pin2* mutant seedling roots still exhibited bending toward darkness. This suggests the involvement of other auxin transporters besides PIN2 in root skototropism. One potential candidate could be the ABCB auxin transporter, which has been shown to play an important role in root phototropism.^15^

Different shade approaches, resulting in reduced light intensity on the dark side of the Petri dish, have different effects on the skototropic response of *Arabidopsis* roots. The use of a black cover creates a complete blockage of light on one side of the Petri dish, providing a clear and distinct contrast between the light and dark conditions. This setup ensures that the roots experience a sharp transition from light to darkness, allowing for a strong skototropic response. The black cover effectively prevents any light leakage and provides a well-defined boundary for the roots. However, the black box induces a strong gradient of light intensity within the Petri dish.^50^ Although the reduced light intensity can influence the strength of the light stimulus perceived by the roots, it may result in a less pronounced skototropic response compared to the black cover setup. Additionally, the presence of some residual light in the dish due to partial blocking may introduce a more gradual transition between light and darkness, potentially affecting the roots’ perception and response. While the distribution pattern of bending angles may differ between the treatments, with the small round Petri dishes with black cover treatment showing a more dispersed pattern than the black box treatment, the overall trend of decreasing skototropism with increasing distance to darkness remains consistent. Moreover, Petri dish shapes may affect factors such as air circulation and humidity within the Petri dish, which can indirectly impact root growth and behavior. Our data show that the round Petri dishes display a more prominent skototropic response compared to the square ones.

Several conclusions were drawn from this study: (1) Plants show root skototropic behavior when they are under light stress conditions. As the distance between seedlings and darkness increases, it becomes more challenging for them to perceive the darkness and exhibit this “escape tropism.” (2) In contrast to the wild-type (Col-0) line, the *atglr3.7 ko* demonstrated a larger percentage of positive root skototropism (bending toward darkness), whereas the *AtGLR3.7 OE* exhibited reverse bending trends, suggesting the AtGLR3.7 may play an important role in root skototropism. (3) The root-positive skototropism of *pin2 knockout* mutants was significantly lower than that of Col-0 seedlings, and there was no noticeable change in root skototropism of *pin2 knockout* mutants under different distance-pattern settings.

Summarily, this study provides valuable insights into the skototropic behavior of *Arabidopsis* roots and the potential involvement of AtGLR3.7 and AtPIN2 in mediating this response. Further studies are needed to elucidate better the underlying molecular mechanisms and signaling pathways involved in root skototropism. Understanding these mechanisms could have implications for improving plant growth and development in various environmental conditions.

## Supplementary Material

Supplemental Material

## Data Availability

The authors declare that all relevant data supporting the findings of this study are available within the paper and its supplementary files. All data for the main figures are provided in Supplementary Data 1. All other data will be available from corresponding authors upon reasonable request.

## Additional information

Supplementary information. For, pre data supporting this study see Supplementary Data 1.
